# Fine-scale genetic structure and genetic diversity in the Chinese crocodile lizard

**DOI:** 10.1016/j.isci.2025.114258

**Published:** 2025-11-27

**Authors:** Guannan Wen, Hongxin Xie, Weiguo Du

**Affiliations:** 1 Institute of Zoology, Chinese Academy of Sciences, Beijing, China; 2 School of Biodiversity, One Health and Veterianry Medicine, College of Medical, Veterinary & Life Sciences, University of Glasgow, Glasgow, UK; 3 State Key Laboratory of Wetland Conservation and Restoration, National Observations and Research Station for Wetland Ecosystems of the Yangtze Estuary, Ministry of Education Key Laboratory for Biodiversity Science and Ecological Engineering, and Institute of Eco-Chongming, School of Life Sciences, Fudan University, Songhu Road 2005, Shanghai 200438, China

**Keywords:** evolutionary biology, genomics, zoology

## Abstract

Population fragmentation and restricted dispersal in endangered species can sharply reduce genetic diversity and adaptive potential, making genetic monitoring vital for conservation. However, whether neutral genetic markers alone can adequately assess adaptive potential remains debated. We examined the fine-scale genetic structure and diversity of the endangered Chinese crocodile lizard (*Shinisaurus crocodilurus*) in three closely located streams: DaChai (DC), YuSan (YS), and ChiShui (CS). Despite their proximity, we found clear genetic differentiation, with DC especially distinct from the CS-YS group. Both subpopulations showed reduced genome-wide diversity and signs of inbreeding. Interestingly, the MHC gene region showed a different pattern. DC individuals had higher MHC diversity, while CS-YS showed signatures of recent selection. These results suggest different evolutionary pressures at immune-related loci in the two subpopulations and highlight the importance of integrating both neutral and functional genetic markers in conservation, as neutral markers alone may overlook key adaptive capacities.

## Highlights

• Fine-scale genetic differentiation was detected among adjacent *S. crocodilurus* streams
• Both subpopulations show reduced genome-wide diversity and inbreeding
• MHC loci reveal contrasting selection patterns between subpopulations
• Integrating neutral and functional markers is essential for conservation management

## Graphical abstract

**Figure:**
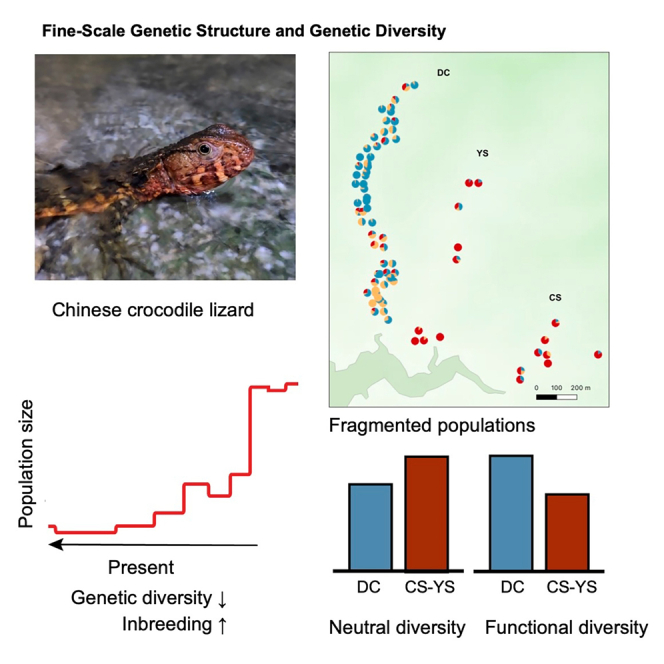

## Introduction

Endangered species often experience population fragmentation and reduced gene flow that increase inbreeding and lead to a decline in genetic diversity. This reduction in genetic variation can compromise the adaptive potential and long-term persistence of a population by limiting its ability to respond to changing environmental conditions. Consequently, accurate assessment of genetic diversity is a fundamental component of population monitoring and conservation management strategies.^1^^,^^2^^,^^3^

In conservation genetics, a long-standing debate centers on the relative utility of neutral versus functional genetic markers as indicators of a population’s overall genetic health.^4^^,^^5^^,^^6^ Neutral markers such as microsatellites and single-nucleotide polymorphisms (SNPs) have been widely applied to infer population structure, estimate gene flow, and reconstruct historical demographic events.^5^^,^^7^ Although these markers provide critical information about the evolutionary history of populations, they do not directly capture the genetic variation that underlies adaptive traits. In contrast, functional genetic variation provides direct insights into adaptive potential. This includes genes involved in immune response, such as the major histocompatibility complex (MHC) as well as stress-response genes like heat shock proteins (HSPs).^8^^,^^9^^,^^10^ However, obtaining comprehensive functional genetic data generally requires extensive genomic resources and ecological context, which are not always available for all species. This discrepancy raises concerns that an overreliance on neutral markers may offer an incomplete picture of genetic health and adaptive capacity. Recent studies emphasize the importance of integrating both neutral and adaptive genetic markers to better understand a population’s long-term survival potential.^11^^,^^12^^,^^13^

The Chinese crocodile lizard (*Shinisaurus crocodilurus*) provides an ideal model for addressing this challenge. As the only survival member of the family Shinisauridae, this semi-aquatic reptile is endemic to southeastern China and northeastern Vietnam. Historically, the species was abundant across the mountain ranges of Guangxi and Guangdong provinces in China.^14^ Due to intense habitat destruction, fragmentation, and illegal collection for the pet trade and traditional medicine, *S. crocodilurus* is listed as endangered on the International Union for Conservation of Nature Red List and is subject to strict international trade regulations under the Convention on International Trade in Endangered Species. Field surveys revealed a drastic population reduction in this species from approximately 6,000 individuals in 1978 to only 950 by 2004 in China,^15^ with at least seven local populations becoming extinct.^15^ Vietnamese populations suffered even greater losses, with fewer than 100 individuals recorded by 2014.^16^ Moreover, genetic studies have revealed that the surviving populations possess relatively low levels of genetic diversity, likely as a consequence of historical bottlenecks and ongoing isolation.^17^^,^^18^^,^^19^^,^^20^

**Figure 1:**
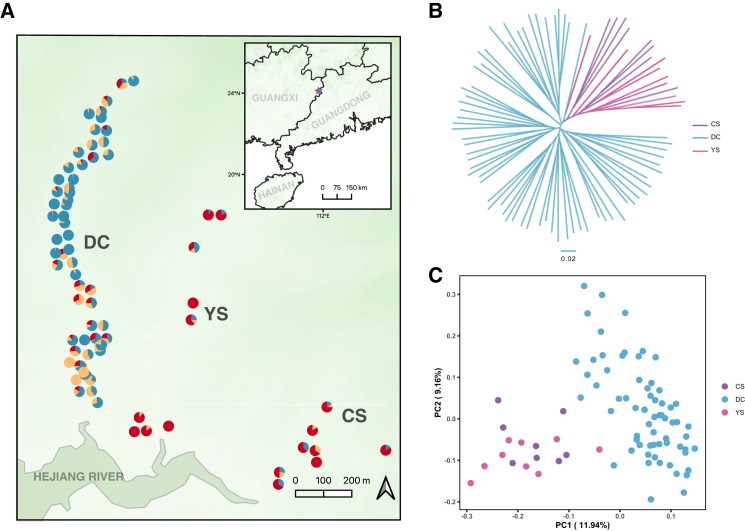
Genetic structure of collected samples (A) The geographic distribution of the sampling locations and the genetic structure inferred using the admixture at *K* = 3, where different colors represent distinct genetic clusters. (B) A neighbor-joining (NJ) tree constructed from the 1-IBS (identity by state) distance matrix, which visualizes the genetic relationships between the individuals. (C) A plot of the first two principal components (PCs) from the PCA, showing the variation in genetic data across the samples.

The Daguishan population, located in the Daguishan National Nature Reserve in Guangxi Province, China, is one of the largest remaining wild populations of *S. crocodilurus*. This population primarily inhabits three adjacent streams: DaChai (DC, 1,750 m, elevation range 86–268 m), YuSan (YS, 1,650 m, range 80–149 m), and ChiShui (CS, 900 m, range 66–181 m) (Figure 1A). The three streams are separated by a maximum distance of roughly 500 m, arranged in a radial pattern, and all drain into the Hejiang River. As a semi-aquatic species, Chinese crocodile lizards are highly dependent on mountain stream habitats and exhibit extremely limited dispersal abilities. Tracking studies reveal strong site fidelity in this species, with an average annual home range of 69 ± 71 m.^21^ In addition, long-term observations suggest that individuals rarely disperse more than 10 m even over a three-year period.^22^ Such movement constraints and habitat specificity could significantly influence genetic diversity patterns within and between populations.^23^^,^^24^^,^^25^ Understanding these genetic patterns is crucial for developing effective conservation strategies, particularly in maintaining genetic connectivity and adaptive potential.

In this study, utilizing whole-genome data obtained from extensive sampling of Daguishan populations of *S. crocodilurus*, we characterized the species’ fine-scale genetic structure and evaluated the effectiveness of neutral and functional genetic markers in assessing its genetic diversity. We used non-coding regions (non-CDS) as representatives of neutral markers and coding regions (CDS) as proxies for functional markers. However, given the limited understanding of the adaptive context for the entire coding genome, we also included the MHC gene, which is a well-characterized functional marker with clear adaptive relevance. We compared genetic diversity metrics derived from the three marker sets to investigate whether neutral markers alone can sufficiently reflect a population’s functional genetic diversity or if incorporating functional markers provides essential additional insights. Ultimately, our goal is to establish a more integrated framework for genetic monitoring and long-term conservation of this endangered species.

## Results

We collected 82 samples from three streams (DC, YS, and CS) within the Guangxi Daguishan Natural Reserve (Figure 1A). The sequencing depth for the individuals ranged from 5.73 to 67.79, covering 62.30%–85.56% of the entire genome (Table S1). After stringent data filtering, we obtained a total of 13,211,431 high-quality SNPs. Kinship analysis detected no first-degree relationships among the sampled lizards (Figure S1).

### Fine-scale population structure

The Daguishan population of wild crocodile lizards exhibited minimal genetic differentiation, as indicated by the star-shaped NJ tree (Figure 1B). At a fine-scale resolution, we found that the Daguishan population can be divided into two colonies. The DC stream exhibited reduced gene flow with the other two streams. In the ADMIXTURE analysis, *K* = 3 provided the best fit (Figure 1A, *K* = 3; Figure S2). Two of the three clusters were specific to the DC stream, separating individuals from the upstream and downstream sections. A third cluster grouped individuals from the CS and YS streams. The principal component analysis (PCA) results further support this pattern (Figure 1C), with PC1 effectively distinguishing the DC stream from the others, while PC2 captures the genetic variation within the DC stream.

### Genetic diversity and inbreeding

Given the observed genetic structure, we further investigated the genetic diversity of the Daguishan population. The overall population exhibited reduced genetic diversity, a characteristic commonly associated with endangered species that have fragmented small populations (Table 1). Its observed heterozygosity (*H*_*o*_) had a median value of 0.192 (95% confidence interval [CI]: 0–0.659), which is significantly lower than the expected heterozygosity (*H*_*e*_), with a median of 0.406 (95% CI: 0.102–0.500). Comparisons between the two identified colonies revealed that the CS-YS colony exhibited slightly higher heterozygosity compared to the DC colony (Table 1, Wilcoxon rank-sum test, *p* < 2e-16).

**Table 1:** Summary of genetic diversity parameters

	DC colony	CS-YS colony	All
*N*	65	17	82
*H*_*e*_	0.383 (0.000, 0.500)	0.360 (0.000, 0.500)	0.406 (0.102, 0.500)
*H*_*o*_	0.193 (0.000, 0.662)	0.222 (0.000, 0.778)	0.143 (0.000, 0.750)
AR	1.370 (1.000, 1.506)	1.370 (1.000, 1.527)	1.390 (1.096, 1.510)

This table presents the median values of key genetic diversity parameters for the DC, CS-YS colony, and the entire Daguishan population, with 95% parametric CIs shown in parentheses. The population size (*N*) for each group was indicated. Parameters include observed heterozygosity (*H*_o_), expected heterozygosity (*H*_e_) and Allelic richness (AR).

**Figure 2:**
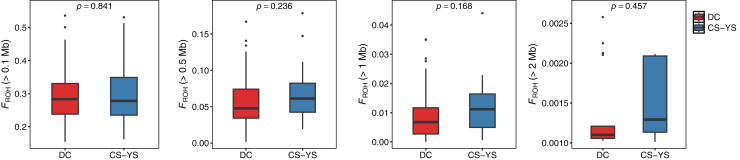
Variation in *F*_*ROH*_ between DC and CS-YS colonies across different ROH length thresholds *F*_*ROH*_ values are shown for ROH length thresholds of 0.1, 0.5, 1, and 2 Mb. *p* values from non-parametric Wilcoxon rank-sum tests are indicated at the top of each panel. Only individuals with ROHs meeting the respective length threshold were included in the *F*_*ROH*_ calculations. The box spans from the 25th to the 75th percentile, with the horizontal line indicating the median value.

Analyses of runs of homozygosity (ROH) were performed using four minimum length thresholds (0.1, 0.5, 1, and 2 Mb) to capture inbreeding signals across different time scales. The proportions of individuals with ROHs above each threshold are shown in Figure S3. All individuals from all populations had ROHs exceeding 0.1 and 0.5 Mb, while only approximately one-third carried long ROHs (>2 Mb), including 29.4% in the CS–YS and 26.2% in DC (Figure S3). Overall *F*_*ROH*_ values showed no significant differences between the two subpopulations across thresholds (Figure 2; Table 2). However, a pattern emerged: *F*_*ROH*_ in DC tended to decline with increasing ROH length thresholds, suggesting reduced recent inbreeding. The lowest *F*_*ROH*_ observed across all individuals was found in DC stream (0.15, at the 0.1 Mb threshold), compared to a mean of 0.30 across all individuals at the same threshold.

**Table 2:** *F*_*ROH*_ values for *Shinisaurus crocodilurus* colonies

Colonies	Mean (SE) *F*_*ROH*_ 0.1 Mb	Mean (SE) *F*_*ROH*_ 0.5 Mb	Mean (SE) *F*_*ROH*_ 1 MB	Mean (SE) *F*_*ROH*_ 2 Mb
DC	0.2949 (0.0806)	0.0569 (0.0344)	0.0099 (0.0088)	0.0014 (0.0005)
CS-YS	0.3039 (0.1075)	0.0700 (0.0425)	0.0125 (0.0107)	0.0015 (0.0005)
Total	0.2968 (0.0862)	0.0596 (0.0363)	0.0105 (0.0092)	0.0014 (0.0005)

Mean *F*_*ROH*_ values (±SE) are shown for each colony (DC, CS-YS) and for the total sample, based on ROH thresholds of 0.1, 0.5, 1, and 2 Mb.

### Historical effective population size dynamics

**Figure 3:**
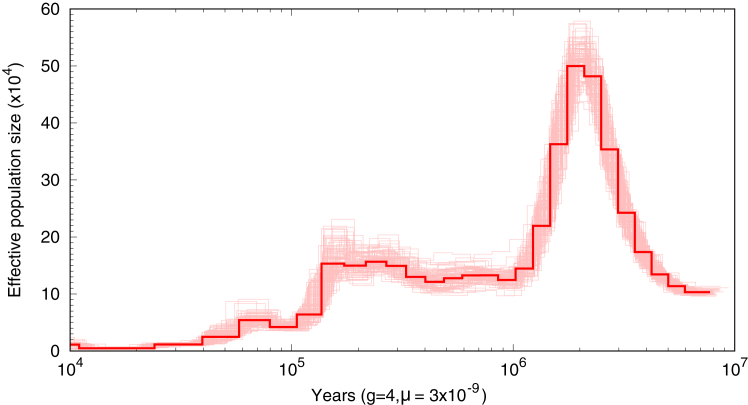
Estimation of historical *N*_*e*_ using the PSMC model An individual with the highest average sequencing coverage was chosen for the analysis. Dashed lines represent 100 bootstraps. Mutation rate was set to 3.05 × 10^−9^ substitutions per site per generation and the generation time is four years.

To better understand the demographic history of the Daguishan population, we inferred historical effective population size (*N*_*e*_) dynamics using the pairwise sequentially Markovian coalescent (PSMC) method.^26^ We recovered a demographic trajectory highly consistent with that reported by Xie et al.,^17^ showing two major periods of population decline in *S. crocodilurus*: the first occurring between ∼2 and 1 million years ago (Mya), and the second between ∼0.2 and 0.1 Mya. Following each decline, the population size dropped to a markedly low level, with the most severe bottleneck occurring after the latter contraction (Figure 3).

### Comparison of neutral and functional genetic diversity

**Figure 4:**
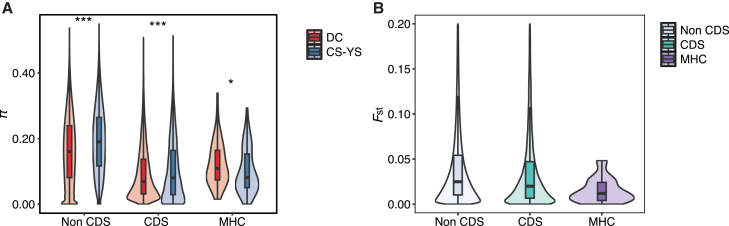
The differences in genetic diversity among the three genomic regions: non-coding (non-CDS), coding (CDS), and MHC gene regions for the DC and CS-YS colonies (A) Genetic diversity was assessed using nucleotide diversity (π), and compared across non-CDS, CDS, and MHC regions using the paired Wilcoxon rank test. Significance levels are indicated (∗*p* < 0.05; ∗∗∗*p* < 0.001). (B) Pairwise comparisons of *F*_ST_ among the three genomic regions were assessed using the Kruskal-Wallis rank-sum test followed by Wilcoxon rank sum post hoc analysis with Benjamini-Hochberg correction (all comparisons, *p* < 0.001). The violin outline reflects the kernel density estimation of the likelihood distribution, while the internal boxplot spans from the 25th to the 75th percentile, with the horizontal line indicating the median value.

**Figure 5:**
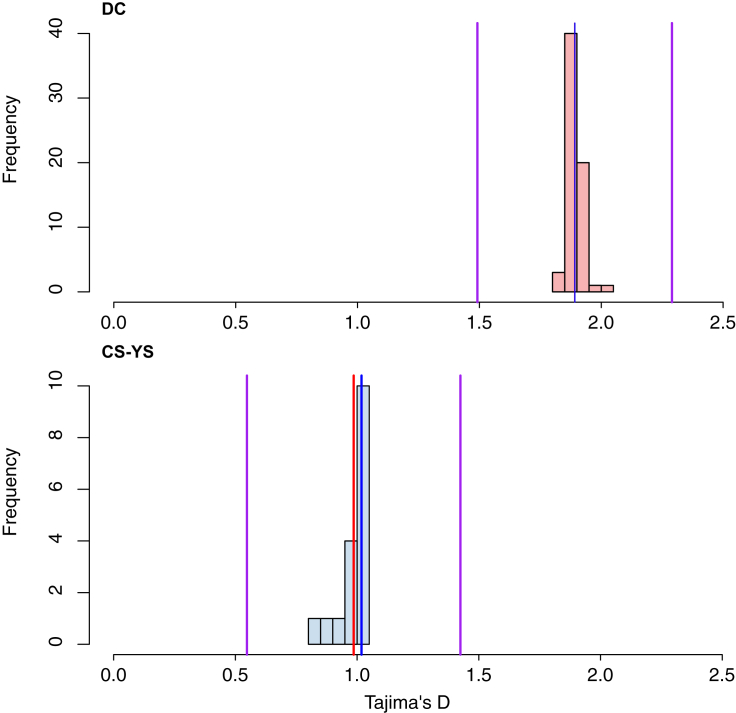
The distribution of Tajima’s *D* values calculated across the MHC gene regions in the DC and CS-YS colonies using Jackknife resampling The red lines are the mean Tajima's D across the Jackknife resampling replicates. The purple lines mark the lower and upper bounds of the 95% confidence interval. The blue lines represent the estimated Tajima’s *D* from the full dataset.

To assess whether neutral genetic diversity can predict functional diversity, we compared nucleotide diversity (*π*) across three genomic regions: non-coding (non-CDS), coding (CDS), and the MHC gene region between DC and CS-YS (Figure 4A). In the non-CDS region, consistent with heterozygosity (*H*_*o*_) data, the DC colony exhibited significantly lower *π* than CS-YS (Wilcoxon rank-sum test, *p* < 0.001). Similarly, the CDS region displayed overall lower diversity compared to the non-CDS region, with DC again showing reduced *π* relative to CS-YS (Wilcoxon rank-sum test, *p* < 0.001). In contrast, the MHC region revealed a higher diversity in DC compared to CS-YS (Wilcoxon rank-sum test, *p* = 0.034). Furthermore, pairwise *F*_ST_ comparisons indicated the lowest differentiation between DC and CS-YS in the MHC region relative to the non-CDS and CDS regions (Figure 4B). Finally, the MHC gene region showed positive Tajima’s *D* in the DC colony (*D* = 1.89, 95% CI: 1.49–2.29) and is higher than that in the CS-YS colony (*D* = 1.02, 95% CI: 0.55–1.42; Figure 5).

**Figure 6:**
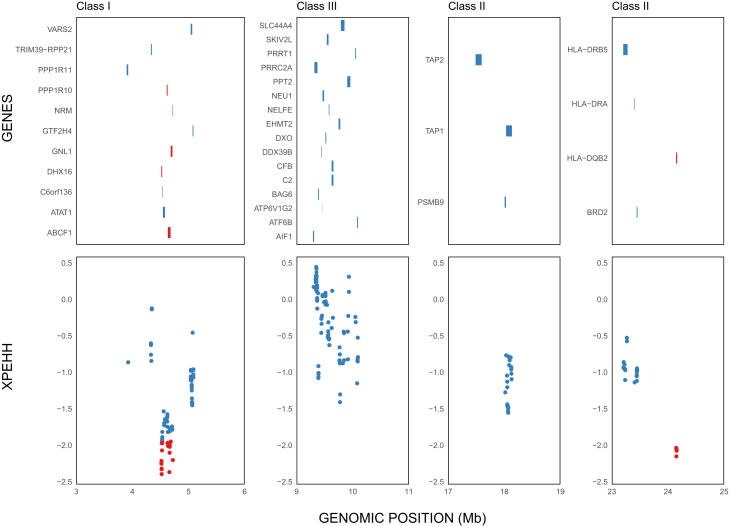
XP-EHH signals and gene annotations cross the MHC region The bottom panel shows XP-EHH values comparing DC against CS-YS colonies. The upper panel displays annotated genes aligned to their genomic positions. Loci with XP-EHH values beyond the top or bottom 5% thresholds (2.10462 and −1.93285, respectively) are highlighted in red.

We examined genetic diversity at the coding regions of individual MHC genes in detail. Of the 30 polymorphic MHC genes identified in the Daguishan population, 23 showed higher diversity in the DC colony compared to the CS-YS colony (Table S3), indicating a bias toward greater diversity in DC (binomial test, *p* < 0.003). Top genes with greatest difference in polymorphism include *HLA-DQB2*, a classic MHC class II gene involved in antigen presentation and adaptive immune response,^27^ as well as non-classical MHC-linked genes such as *PPP1R1* (a E3 ubiquitin-protein ligase that functions in defense response to Gram-positive bacterium)^28^ and PSMB9 (an immunoproteasome subunit important for antigen processing to generate class I binding peptides).^29^ Selection analyses further identified two sets of loci located in both MHC class I and class II regions with significant negative Cross-population extended haplotype homozygosity (XP-EHH) values (within the extreme 5% tail of the genome-wide distribution), indicating recent positive selection in the CS-YS colony (Figure 6). These loci encompassing six MHC genes, five of which also showed higher diversity in the DC colony. The exception is *GNL1*, which had no polymorphic sites in its coding region.

## Discussion

Our study reveals that even within a geographically restricted area, the endangered Chinese crocodile lizard exhibits notable genetic substructure. Despite the streams being only a few hundred meters apart, individuals from the DC colony are genetically distinct from those in the CS-YS colony. Even within the DC stream itself (approximately 1,600 m in length), there is a detectable substructure between upstream and downstream sections. This fine-scale differentiation likely reflects the species’ semi-aquatic lifestyle and strong site fidelity,^22^ which constrain movement even across short distances. The reduced gene flow may promote genetic fragmentation and increase the risk of inbreeding, ultimately threatening the species’ long-term adaptive potential.^30^^,^^31^^,^^32^

Inbreeding level assessed by *F*_*ROH*_ supports a long-term inbreeding history in crocodile lizards. Moreover, the CS-YS colony, which has a smaller population size than the DC colony, shows signs of more intense recent inbreeding. Generally, the observed mean *F*_*ROH*_ in our dataset (0.30, at the 0.1 Mb threshold) is comparable to other endangered animal species like the mountain and eastern lowland gorillas (34.5% and 38.4%).^33^ But considerably less severe than that of some extremely inbred species like the brown-eared pheasant (>80%).^31^ Demographic analyses indicate that *S. crocodilurus* experienced a severe historical bottleneck and currently maintains an extremely low population size. This historical contraction is consistent with earlier work demonstrating that prolonged bottlenecks may lead to both reduced neutral diversity and an increased risk of inbreeding depression.^17^ Although genetic purging can act to remove highly deleterious alleles during such contractions, our results show that genetic heterozygosity *H*_*o*_ is considerably lower than *H*_*e*_, suggesting that inbreeding due to small population size is leading expedited purging.^32^^,^^34^ The limited genetic variation of the current population may impair the species’ ability to adapt to ongoing global climate change.^35^

Our results revealed a contrasting pattern of genetic diversity across genomic regions: while both non-coding and coding regions exhibit reduced diversity in the DC colony, the MHC region displays elevated diversity compared to CS-YS. The contrasting patterns observed at MHC genes between the CS-YS and DC colonies suggest that local selection or drift acting specifically on immune genes may have driven elevated differentiation at these loci. The MHC genes in DC may be under more stable, long-term balancing selection, whereas the reduced diversity and directional selection signals in CS-YS could reflect recent shifts in selective pressures. More specifically, Tajima’s *D* values were positive in both populations, suggesting the presence of balancing selection at broader timescales. However, the reduced diversity and strong selection signals in CS-YS point to recent selective sweeps potentially reducing allelic variation. The role of balancing selection in maintaining adaptive variation at immune-related loci even in the face of overall loss of neutral diversity has long been reported in other endangered species.^36^^,^^37^^,^^38^ In light of several pathogen-caused diseases have been reported in this species,^39^^,^^40^ preserving high MHC diversity may therefore be critical for the long-term survival of its fragmented populations.^8^^,^^9^^,^^37^

Although these subpopulations are separated by only a few hundred meters and they are unlikely to have originated from a different ancestral gene pool (as suggested by the very low genome-wide *F*_ST_, Figure 4B; also see^17^), the observed differentiation at functionally distinct loci is somewhat unexpected. A relevant comparison can be found in *Egernia stokesii*, a species with ecological characteristics similar to *S. crocodilurus*, including highly fragmented habitats and extremely limited dispersal ability. Pearson et al.^41^ showed that populations displayed clear genetic structure at neutral loci, but minimal divergence at MHC loci. They attributed this pattern to stabilizing or convergent selection on immune genes, which can override local drift when environmental conditions are similar and some gene flow persists. In contrast, we found elevated divergence at MHC loci, probably driven by recent selection in the CS-YS colony. This suggests that while balancing selection may maintain MHC polymorphism in some subpopulations (e.g., DC), localized or recent directional selection may drive divergence in others. These findings highlight that MHC loci can deviate from genome-wide patterns in both directions (toward reduced or enhanced divergence) depending on the balance between drift, gene flow, and spatially variable selection, reinforcing the complexity of immune gene evolution at fine spatial scales.

From a conservation perspective, the observed patterns provide a rational for implementing more fine-scale, population-specific management approaches. For the CS-YS colony, reduced MHC diversity and signs of recent directional selection raise concerns about a potential erosion of adaptive capacity. While the underlying drivers remain unclear, the narrowing of functional diversity may compromise long-term population health. Particularly under future environmental shifts or emerging disease threats, limited immunogenetic variation could constrain adaptive responses.^9^^,^^42^ Although the DC colony currently maintains a larger population size and higher MHC diversity compared to CS-YS, it may still face important risks. First, the potential accumulation of inbreeding over time, even in large populations, can erode genetic health, particularly under restricted gene flow.^6^^,^^9^^,^^43^ Second, unchecked population growth may surpass the local environmental carrying capacity, increasing the likelihood of demographic collapse due to resource limitation or disease outbreaks.^44^^,^^45^ These findings suggest an urgent need for targeted genetic monitoring^9^ and pathogen surveillance^46^ to assess immunogenetic health and identify emerging disease risks. Where appropriate, managed gene flow^20^^,^^47^ may also be considered as a tool to preserve the evolutionary resilience of this vulnerable subpopulation.

In summary, the Chinese crocodile lizard faces population fragmentation and inbreeding. However, the maintenance of functional diversity, particularly at MHC loci, may buffer against the potential environmental and pathogenic challenges. A conservation framework that includes both neutral and functional genetic data is essential to safeguard the species’ adaptive potential. Our findings support growing evidence that functional diversity, especially at immune-related loci, can be decoupled from neutral diversity.^6^^,^^48^^,^^49^ This highlights its distinct role in promoting long-term species viability. Future studies should examine other populations to determine whether similar patterns occur across the species’ range.

### Limitations of the study

First, although our sampling effort was extensive in both sample size and spatial coverage, it was still limited to a few adjacent streams, which constrains inference about broader population processes across the species’ range. Second, while whole-genome resequencing offers high resolution, our ability to disentangle the relative effects of demography, drift, and selection is restricted by the absence of temporal data and environmental or pathogen profiling. Third, the detection of selection at MHC loci is based on statistical signatures (e.g., XP-EHH, Tajima’s *D*) rather than direct functional assays, so adaptive interpretations should be viewed as provisional. Finally, integrating ecological, behavioral, and long-term monitoring data will be essential to link genomic patterns to demographic trends and fitness outcomes in future work.

## Resource availability

### Lead contact

Requests for further information and resources should be directed to and will be fulfilled by the lead contact, Du Weiguo (duweiguo@fudan.edu.cn).

### Materials availability

This study did not generate new unique reagents.

### Data and code availability

• The raw sequencing data from this study has been deposited in the National Genomics Data Center (NGDC, https://ngdc.cncb.ac.cn) under BioProject: PRJCA048623.
• This study did not generate new codes.
• No additional information available.

## Acknowledgments

We thank L.S., Y.S., and L.Z. for their assistance with field sampling. We thank Daguishan National Nature Reserve for providing permission and support for field work. This research was supported by the 10.13039/501100012166National Key R&D Program of China (no. 2023YFF1304800).

## Author contributions

W.G. and X.H. contributed equally to this work, including sampling, SNP calling, population genomic analyses, and manuscript preparation; D.W. designed the study, secured funding, and refined the manuscript.

## Declaration of interests

The authors declare no competing interests.

## Declaration of generative AI and AI-assisted technologies in the writing process

During the preparation of this work, the authors used ChatGPT (OpenAI) to assist with language editing, grammar refinement, and improving the clarity and flow of the manuscript. After using this tool, the author(s) carefully reviewed and edited the content to ensure accuracy and take(s) full responsibility for the final version of the publication.

## STAR★Methods

### Key resources table

**Table:** 

REAGENT or RESOURCE	SOURCE	IDENTIFIER
**Critical commercial assays**		

AllPure DNA Kit	CWBIO	CW0591S

**Deposited data**		

Sequencing Data	This study	Genome Sequence Archive (GSA)-Bioproject: PRJCA048623

**Software and algorithms**		

fastp v.0.23.4	Chen et al.^50^	https://github.com/OpenGene/fastp.git
BWA-MEM v0.7.18	Vasimuddin et al.^51^	https://github.com/lh3/bwa.git
SAMtools v1.18	Li et al.^52^	https://github.com/samtools/samtools.git
Picard v3.3.0	Broad Institute^53^	https://github.com/broadinstitute/picard.git
GATK v4.2.6.1	Van der Auwera et al.^54^^,^^55^	https://github.com/broadinstitute/gatk.git
SNPable	Li^56^	https://github.com/lh3/misc
Bcftools v1.18	Danecek et al.^57^	https://github.com/samtools/bcftools.git
VCFtools v0.1.15	Danecek et al.^58^	https://github.com/vcftools/vcftools.git
PLINK v1.90b3.46	Purcell et al.^59^	https://github.com/chrchang/plink-ng.git
KING	Manichaikul et al.^60^	https://kingrelatedness.com/
ADMIXTURE v1.3.0	Alexander and Lange^61^	https://dalexander.github.io/admixture/
Pophelper	Francis^62^	https://github.com/royfrancis/pophelper.git
R 4.3.1	R. Core Team^63^	https://www.r-project.org/
PSMC	Li and Durbin^26^	https://github.com/lh3/psmc.git
genomics_general	Martin^64^	https://github.com/simonhmartin/genomics_general.git
selscan v2.0.0	Szpiech^65^	https://github.com/szpiech/selscan.git

### Experimental model and study participant details

The *Shinisaurus crocodilurus* populations in this study were sampled from the Daguishan National Nature Reserve, Guangxi Province, China (The Daguishan population, 24.10° N, 111.81° E). Saliva samples were collected from 82 individuals across three streams: ChiShui (CS), YuSan (YS), and DaChai (DC). Specifically, 8 samples were obtained from CS, 9 from YS, and 65 from DC (Table S1).

### Method details

#### Whole-genome sequencing and data processing

Genomic DNA was extracted using the AllPure DNA Kit (CWBIO), a method effective for isolating DNA from saliva samples. Subsequently, whole-genome sequencing was performed on the DNBSEQ-T7 platform. We adopted a cost-effective sequencing strategy by selecting 8 individuals from the three streams (4 from DC, 3 from YS, and 1 from CS) for high-depth whole-genome sequencing at ∼30×, and the remaining individuals were sequenced at a lower depth of ∼5×.

Raw sequencing data underwent initial quality control using fastp^50^ to eliminate contaminants and trim low-quality read ends. The cleaned reads were aligned to a chromosome-level reference genome^17^ employing BWA-MEM.^51^ Subsequent processing, including file format conversion and sorting, was performed with SAMtools.^52^ To ensure data accuracy, PCR duplicates were identified and removed using Picard tools.^53^ SNP genotyping was conducted with GATK's HaplotypeCaller.^54^^,^^55^

To maximize the reliability of the identified SNPs, a stringent filtering protocol was applied: Firstly, genotypes for all samples were determined using GATK's CombineGVCFs and GenotypeGVCFs. Then, focusing on the eight high-depth samples, SNPs were filtered based on the following criteria: 1) keep biallelic SNPs with sequencing depths between 5 and 600; 2) exclude loci with a minor allele frequency (MAF) less than 0.0000001 and Hardy-Weinberg equilibrium (HWE) *p*-values below 1e-50 to minimize potential errors; 3) keep loci with genotype quality (GQ) scores exceeding 30; 4) exclude loci exhibiting more than 10% missing genotypes; 5) remove individuals with over 30% missing data. The high-quality SNPs identified from the high-depth samples were subsequently extracted from the genotypes of the low-depth samples. Additionally, repetitive genomic regions were masked using Heng Li’s SNPable program,^56^ and only chromosomal sequences were retained for further analysis. Filtering and data manipulation were executed utilizing a combination of Bcftools,^57^ VCFtools,^58^ and PLINK,^59^ adhering to standard practices in population genomics.

#### Population structure and genetic diversity

To analyze population structure and genetic diversity, additional filtering steps were applied. SNPs located within protein-coding genes and their 1 kb flanking regions were excluded, and only SNPs with a MAF > 0.05 were retained. Linkage disequilibrium (LD) pruning was performed using a sliding window of 50 SNPs (advancing 10 SNPs per step) with an *r*^*2*^ threshold of 0.2, randomly removing one SNP from each linked pair. After these filtering steps, a total of 209,456 high-quality SNPs were retained for downstream analyses.

Pairwise relatedness was estimated using KING.^60^ The kinship coefficient for the 82 individuals was inferred from whole-genome SNPs. The estimated kinship coefficient ranges were as follows: [>0.354] for monozygotic twins, [0.177, 0.354] for first-degree relationships, [0.0884, 0.177] for second-degree relationships, and [0.0442, 0.0884] for third-degree relationships. No samples showed relatedness greater than second-degree.

The neighbor-joining (NJ) tree was constructed based on a 1-IBS (identity-by-state) matrix calculated using PLINK, and PCA was also performed using PLINK. For admixture analysis, ADMIXTURE^61^ was run for *K* values ranging from 1 to 6 with 50 replicates (random seed), with convergence assessed using the default termination criterion (*ε* = 10^−4^). The run with the highest log-likelihood at each *K* was visualized using the R package ‘pophelper’,^62^ and clustering solutions were validated by plotting all runs within the top 10% of log-likelihood scores. Genetic diversity metrics, including pairwise *F*_ST_, *H_o_*, and *H_e_*, were calculated using VCFtools.

We used multiple ROH length thresholds (0.5, 1, and 2 Mb) as ROHs of different lengths capture inbreeding signals from different time scales. Shorter ROHs typically reflect older inbreeding events or historical background relatedness, whereas longer ROHs indicate more recent inbreeding due to endogamy or small effective population size. The inbreeding coefficient *F*_*ROH*_ proposed by McQuillan et al.^66^ was used, which defined as the total length of all of an individual's ROHs /the length of the autosomal genome covered by SNPs. By comparing *F*_*ROH*_ across multiple thresholds, we can disentangle the contributions of historical versus recent processes shaping inbreeding patterns.

Given the unequal sample sizes across groups, non-parametric Wilcoxon rank sum tests were used for all pairwise comparisons, with *p*-values adjusted using the Benjamini-Hochberg method in R.^63^

#### Demographic history

Historical effective population size dynamics were inferred using the pairwise sequentially Markovian coalescent model (PSMC).^26^ The individual with the highest sequencing depth (dx05) was used for better demographic estimation. The read mapping BAM file was used to obtain the psmcfa file required for PSMC. Variant calling was done using a two-step process of “bcftools mpileup” and “bcftools call”. “vcfutils.pl vcf2fq” command from bcftools package was then used to transform the VCF file to diploid consensus sequences with the option “-d 15 -D 100 -l 5” to allow for minimum depth of 15, maximum depth of 100 (∼2× average depth), and filtering of sites within 5 bp of an indel. “fq2psmcfa” command from PSMC was then used to transform the sequence to psmcfa format using “-q 20” for filtering genotype quality. Bootstrap data was obtained using “splitfa” command from PSMC. Parameters for PSMC analysis were set to “-N25 -t15 -r5 -p “2+2+25∗2+4+6”” because the default “-p 4+25∗2+4+6” can potentially lead to a recent artificial peak, which was illustrated in a recent study.^67^ PSMC was run for the whole dataset and 100 bootstraps were run by adding the “-b” option. Finally, mutation rate was set to 3.05 × 10^−9^ substitutions per site per generation and a generation time of 4 years were used. Both parameters were based on published estimates.^17^^,^^22^

#### Comparison analysis of genomic diversity

Population genetic statistics, *π* and *F*_ST_, were calculated for the two main colonies, DC and CS-YS, based on the non-CDS, CDS, and the CDS of MHC genes. Non-CDS region SNPs were identified by excluding sites within the CDS and the 1 kb flanking region of a CDS. The MHC gene list was obtained from the genome annotation of a previous study^68^ (Table S2). Population genetic statistics were computed using “popgenWindows.py” from the ‘genomics_general’ toolkit,^64^ applying a 20-site sliding window with a 5-site step. Differences in medians across multiple groups were assessed using the Kruskal-Wallis rank sum test, with post hoc pairwise comparisons tested using the Wilcoxon rank sum test and the Benjamini-Hochberg *p*-value adjustment method. All analyses were conducted in R,^63^ with statistical significance at *p* < 0.05. Tajima’s *D* in the MHC gene region was calculated using a custom R script combining all MHC gene SNPs. The 95% confidence intervals for Tajima’s *D* in the DC and CS-YS colonies were estimated using a jackknife resampling approach, which involved iteratively removing one sample at a time to recalculate Tajima’s *D*.

Cross-population extended haplotype homozygosity (XP-EHH) analyses were performed for all polymorphic sites across the genome using selscan v2.0.0.^65^ The resulting XP-EHH scores were then normalized using the ‘--crit-percent’ parameter set to 0.05, identifying the top and bottom 5% of the empirical distribution as outliers for candidate selection signals.

### Quantification and statistical analysis

All statistical analyses were performed using R.^63^ Statistical details, including sample sizes (N), definitions of N, statistical tests, and measures of center and dispersion, are reported in the figure legends, table captions, and results.

Genetic diversity metrics (*H_o_*, *H_e_*, AR) were summarized as medians with 95% parametric confidence intervals for each colony (Table 1). *F*_*ROH*_ was quantified across ROH length thresholds (0.1–2 Mb) and summarized as mean ± SE (Table 2). Comparisons of *F*_*ROH*_ between colonies were performed using Wilcoxon rank-sum tests, with *P*-values shown in Figure 2. Differences in nucleotide diversity (*π*) among genomic regions (non-CDS, CDS, MHC) were assessed using the paired Wilcoxon rank test (Figure 4). Pairwise *F*_ST_ comparisons among regions were assessed using the Kruskal–Wallis test, followed by Wilcoxon rank-sum post hoc tests with Benjamini–Hochberg correction (Figure 4). Tajima’s *D* for MHC regions was estimated using Jackknife resampling, with mean values and 95% confidence intervals reported (Figure 5).
