# Supplementary material for: Fine-scale genetic structure and genetic diversity in the Chinese crocodile lizard
Source: iScience. 2025 Nov 27;29(1):114258. doi: 10.1016/j.isci.2025.114258 (PMC12757535; doi:10.1016/j.isci.2025.114258)
Supplement: Document S1. Figures S1–S3 and Tables S1–S3 [file mmc1.pdf]

iScience, Volume *29*

Supplemental information

Fine-scale genetic structure

and genetic diversity

in the Chinese crocodile lizard

Guannan Wen, Hongxin Xie, and Weiguo Du

Supplementary figures

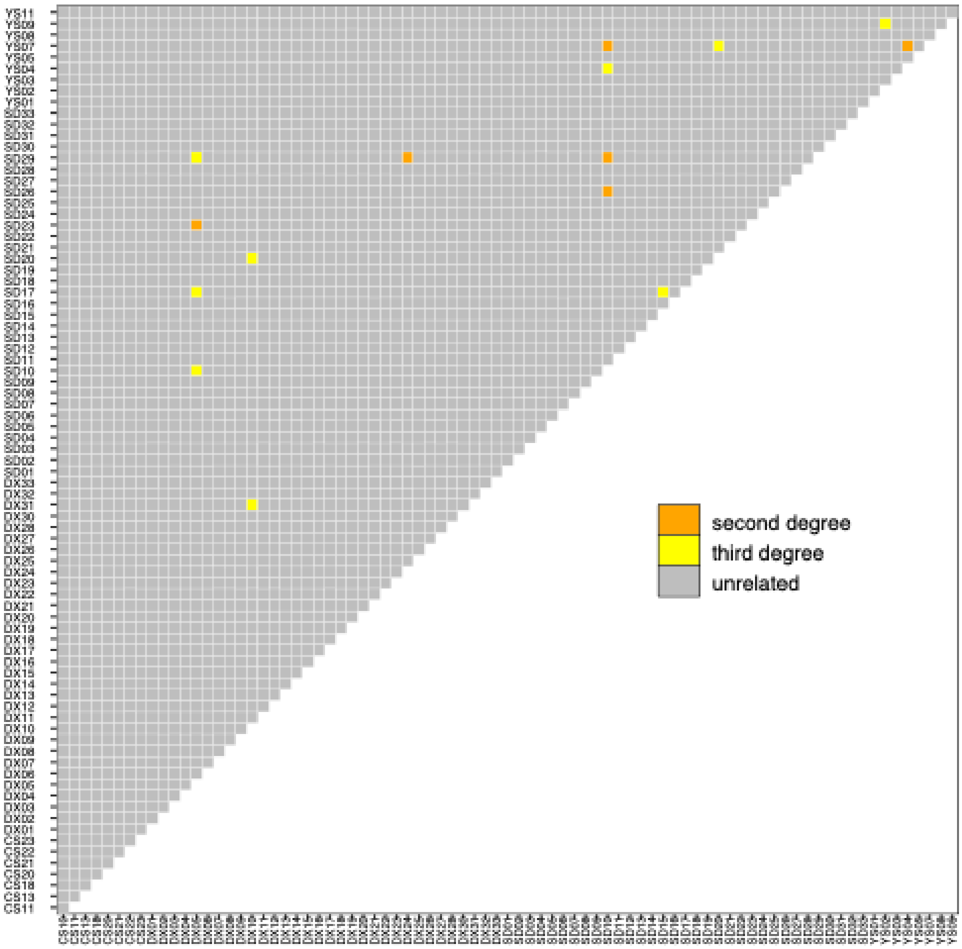

Figure S1. The Kinship Matrix for 82 *S. crocodilurus* Lizards. A heatmap representation of pairwise kinship coefficients among 82 wild *S. crocodilurus* individuals, estimated using whole-genome SNPs. Darker colors indicate lower relatedness, while lighter colors represent higher relatedness.

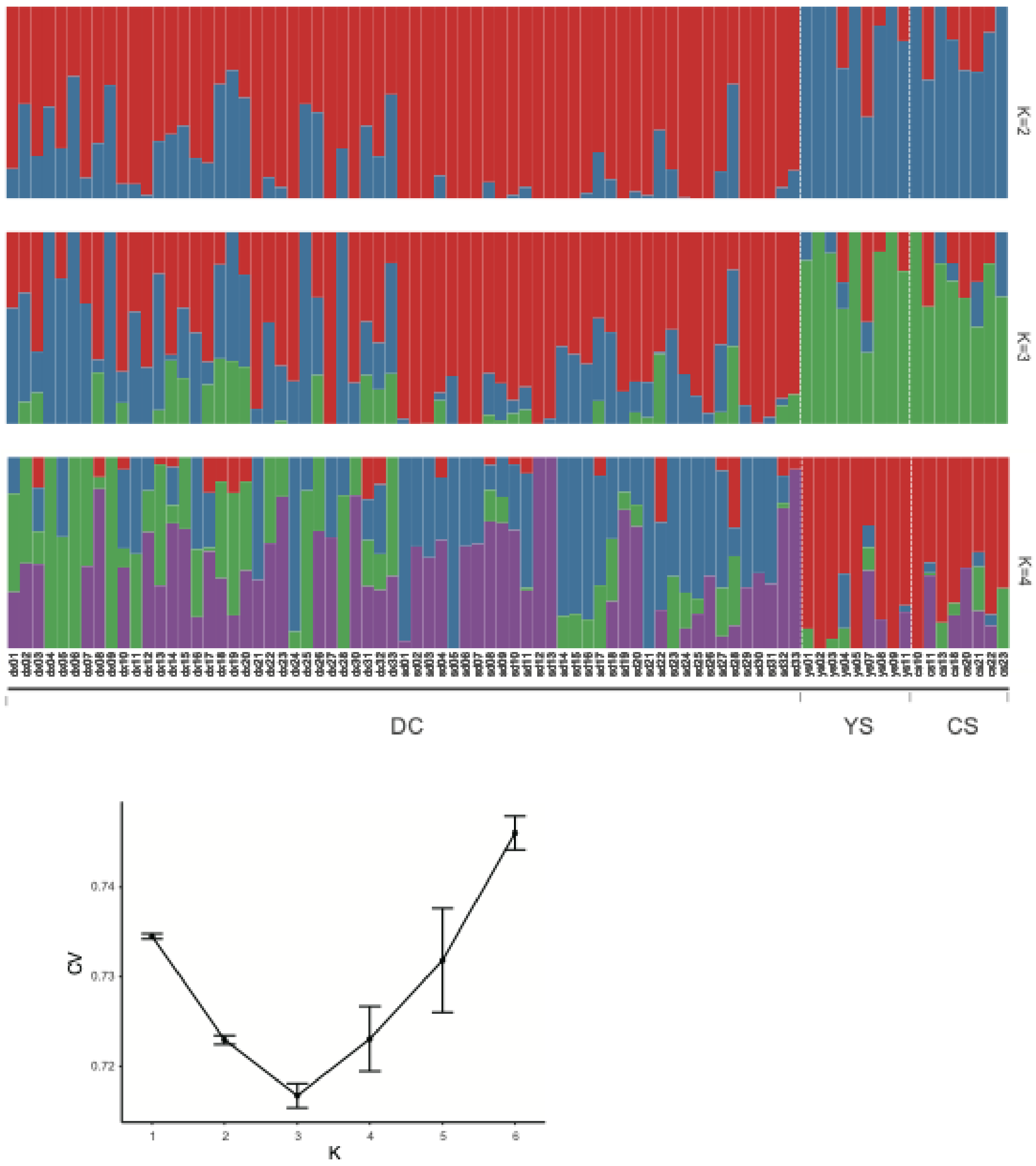

Figure S2. The Admixture Plots and CV Values from K = 2 to 4. Admixture bar plots showing individual ancestry proportions at different assumed population clusters (K = 2 to 4). The cross-validation (CV) error values for each K are also provided to assess model fit and determine the most likely number of genetic clusters.

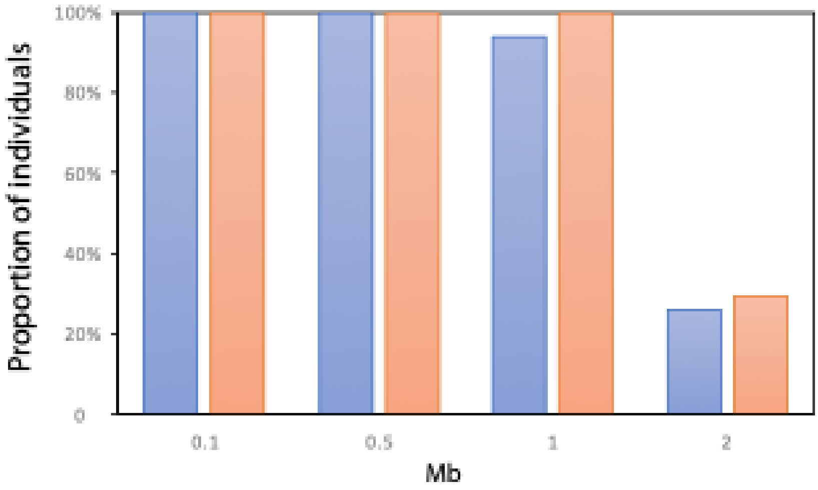

Figure S3. Proportion of individuals with ROHs above given minimum length thresholds. The proportion of individuals carrying at least one ROH > 0.1, 0.5, 1, or 2 Mb is shown for the DC and CS-YS colonies.

Supplementary tables

Table S1. Sample and Sequencing Information for 82 *S. crocodilurus* Lizards. An overview of the sequencing data for each of the 82 wild *S. crocodilurus* individuals. It includes sample IDs, locations, total reads, percentage of mapped reads, genome coverage, and final sequencing depth.

ID	Sex	Age	Location	No. total reads	Mapped Rates (%)	Average Coverage (%)	Mean Depth
cs10	F	subadult	CS	71389062	98.36	67.46	6.56
cs11	M	subadult	CS	101502568	93.38	72.61	9.46
cs13	-	juvenile	CS	84076310	98.39	71.53	7.81
cs18	F	adult	CS	91783914	97.33	70.18	8.21
cs20	F	adult	CS	69886614	98.69	67.47	6.66
cs21	M	adult	CS	437755234	98.01	83.14	38.45
cs22	F	subadult	CS	100010426	93.60	70.93	8.31
cs23	F	juvenile	CS	96679030	98.17	73.40	9.72
dx01	-	juvenile	DC	74153880	94.73	66.57	7.07
dx02	-	juvenile	DC	69858686	91.81	64.97	6.97
dx03	M	subadult	DC	104854698	98.21	72.63	9.45
dx04	-	juvenile	DC	93737162	97.05	71.16	8.73
dx05	F	adult	DC	872724372	99.27	85.56	67.79
dx06	F	adult	DC	70977710	92.34	65.73	6.38
dx07	-	juvenile	DC	94348242	86.26	69.46	7.63
dx08	-	adult	DC	75693896	83.05	65.20	6.61
dx09	M	juvenile	DC	66654112	92.82	63.57	6.06
dx10	M	adult	DC	118421640	96.28	73.31	9.57
dx11	F	juvenile	DC	108800522	96.52	71.70	10.07
dx12	F	juvenile	DC	71344914	94.65	66.28	5.91
dx13	M	juvenile	DC	84853960	98.18	68.74	7.32
dx14	M	juvenile	DC	71090058	98.11	65.91	6.09
dx15	M	juvenile	DC	78269294	98.91	68.67	6.98
dx16	F	adult	DC	587415066	93.81	83.65	42.18
dx17	M	adult	DC	107059810	91.41	70.43	8.62
dx18	F	adult	DC	116586114	89.27	73.21	10.32
dx19	-	juvenile	DC	109484884	89.76	71.86	9.36
dx20	F	juvenile	DC	69437390	92.54	65.57	6.47
dx21	M	subadult	DC	102833976	95.52	70.40	8.88
dx22	-	juvenile	DC	96122322	97.54	71.72	8.72
dx23	F	adult	DC	74092228	98.67	68.50	8.25
dx24	F	adult	DC	100195686	80.81	69.03	7.45
dx25	F	juvenile	DC	66602202	98.28	66.35	6.19
dx26	F	adult	DC	89186582	92.23	68.46	7.20
dx27	M	subadult	DC	92429290	96.86	69.71	7.87
dx28	F	adult	DC	85441876	98.43	68.53	8.09

ID	Sex	Age	Location	No. total reads	Mapped Rates (%)	Average Coverage (%)	Mean Depth
dx30	M	juvenile	DC	91449366	98.58	70.66	9.22
dx31	M	juvenile	DC	130463140	99.00	74.23	12.48
dx32	-	juvenile	DC	76556540	95.72	67.84	7.65
dx33	F	adult	DC	74312422	94.17	66.50	7.21
sd01	F	juvenile	DC	135658182	98.53	74.52	14.78
sd02	M	juvenile	DC	83386516	87.68	66.56	7.43
sd03	M	juvenile	DC	80194924	82.12	65.12	6.86
sd04	F	adult	DC	67727078	96.24	65.10	5.87
sd05	F	juvenile	DC	75109442	92.90	66.40	7.54
sd06	M	subadult	DC	77971386	86.48	64.39	6.73
sd07	F	juvenile	DC	75417588	94.21	66.52	7.43
sd08	F	juvenile	DC	73841728	92.64	66.07	7.35
sd09	F	juvenile	DC	72456596	91.71	65.92	8.27
sd10	M	adult	DC	404345958	96.49	82.32	32.95
sd11	F	adult	DC	87320886	92.46	68.45	7.85
sd12	M	adult	DC	69533122	85.11	62.30	5.73
sd13	M	subadult	DC	94676124	95.38	70.35	8.49
sd14	M	juvenile	DC	79973580	92.12	67.58	6.76
sd15	M	adult	DC	113384138	90.04	72.25	9.28
sd16	F	adult	DC	72068524	93.13	66.51	6.28
sd17	F	subadult	DC	110557290	94.33	72.72	10.63
sd18	F	juvenile	DC	69218264	98.13	67.22	6.30
sd19	F	juvenile	DC	106184714	96.05	72.03	9.16
sd20	M	subadult	DC	123700210	96.63	74.01	10.78
sd21	F	juvenile	DC	75678764	98.89	67.98	6.91
sd22	M	juvenile	DC	95805992	97.91	71.55	8.63
sd23	F	juvenile	DC	95972460	96.85	71.43	8.33
sd24	M	adult	DC	94135582	93.02	70.87	8.26
sd25	M	adult	DC	67965606	97.01	67.03	6.15
sd26	M	adult	DC	108802884	95.33	73.00	9.36
sd27	F	juvenile	DC	96702018	96.69	71.46	8.84
sd28	M	adult	DC	102293118	97.70	72.89	9.56
sd29	M	adult	DC	452070646	97.28	83.63	39.40
sd30	-	juvenile	DC	67941390	91.22	65.33	6.63
sd31	-	juvenile	DC	99233256	91.10	70.13	10.41
sd32	-	juvenile	DC	70258742	95.84	66.10	6.75
sd33	-	juvenile	DC	84418656	96.38	70.05	7.57
ys01	M	subadult	YS	81879748	97.37	68.24	7.89
ys02	F	adult	YS	439057774	98.71	82.75	39.43
ys03	-	juvenile	YS	105109456	90.80	71.60	9.43
ys04	-	juvenile	YS	318803740	91.35	80.89	26.23
ys05	-	subadult	YS	89960406	94.24	68.48	7.70
ys07	F	adult	YS	432619710	89.53	82.35	32.95
ys08	-	juvenile	YS	85694602	95.36	69.58	7.66
ys09	-	juvenile	YS	120689770	91.89	72.76	10.74
ys11	-	juvenile	YS	74592954	96.74	68.80	6.08

Table S2. The Genomic Information for MHC Genes of *S. crocodilurus* Lizards. A summary of MHC genes identified in the *Shinisaurus crocodilurus* genome, including gene IDs, chromosomal locations, and predicted functional annotations.

Protein	Class	Gene ID	Chromosome	Start	End
GNL1	Class I	evm.model.scaffold_277.191	7	4682499	4712213
VARS2	Class I	evm.model.scaffold_277.200	7	5039088	5066172
PPP1R11	Class I	evm.model.scaffold_277.160	7	3897266	3924096
ATAT1	Class I	evm.model.scaffold_277.186.2	7	4543908	4578537
PPP1R10	Class I	evm.model.scaffold_277.189	7	4611084	4627220
ABCF1	Class I	evm.model.scaffold_277.190	7	4631861	4679426
TRIM39-RPP21	Class I	evm.model.scaffold_277.175	7	4329924	4345600
GTF2H4	Class I	evm.model.scaffold_277.201.1	7	5072557	5086816
C6orf136	Class I	evm.model.scaffold_277.185	7	4530984	4538940
NRM	Class I	evm.model.scaffold_277.192	7	4716155	4722635
DHX16	Class I	evm.model.scaffold_277.184	7	4515753	4530049
TAP2	Class II	evm.model.scaffold_14.1211	7	17489940	17597781
HLA-DRB5	Class II	evm.model.scaffold_14.1102	7	23202134	23279308
HLA-DRA	Class II	evm.model.scaffold_14.1097	7	23399695	23408109
PSMB9	Class II	evm.model.scaffold_14.1201	7	18007086	18031031
TAP1	Class II	evm.model.scaffold_14.1200	7	18033526	18136550
HLA-DQB2	Class II	evm.model.scaffold_14.1074	7	24144569	24161997
BRD2	Class II	evm.model.scaffold_14.1096.4	7	23441549	23456133
DDX39B	Class III	evm.model.scaffold_353.218	7	9436610	9445854
SLC44A4	Class III	evm.model.scaffold_353.203	7	9789971	9853235
EHMT2	Class III	evm.model.scaffold_353.205	7	9748300	9777432
DXO	Class III	evm.model.scaffold_353.213.6	7	9513691	9527773
ATP6V1G2	Class III	evm.model.scaffold_353.217	7	9452727	9455716
AIF1	Class III	evm.model.scaffold_353.227	7	9291471	9312333
PRRT1	Class III	evm.model.scaffold_353.198	7	10044878	10057595
SKIV2L	Class III	evm.model.scaffold_353.211	7	9537942	9568589
PRRC2A	Class III	evm.model.scaffold_353.226.5	7	9316941	9371028
NEU1	Class III	evm.model.scaffold_353.216	7	9462106	9488762
BAG6	Class III	evm.model.scaffold_353.224	7	9381973	9397901
NELFE	Class III	evm.model.scaffold_353.210	7	9573886	9584662
C2	Class III	evm.model.scaffold_353.207	7	9623425	9656560
CFB	Class III	evm.model.scaffold_353.207	7	9623425	9656560
ATF6B	Class III	evm.model.scaffold_353.196	7	10078268	10094045
PPT2	Class III	evm.model.scaffold_353.200	7	9902848	9956925

Table S3. Genetic diversity of MHC genes in the DC and CS–YS colonies. Mean nucleotide diversity (π) was calculated for SNPs located within the coding regions (CDS) of each MHC gene. Only genes with at least one heterozygous site were included. "delta_pi" denotes the difference in diversity between colonies (π_DC – π_CS-YS), and "log2_ratio" represents the log-transformed fold change in diversity (log_2(π_DC / π_CS-YS)).

Protein	GeneID	site n	pi dc	pi cs_ys	delta pi	log2 ratio
PPP1R11	evm.model.scaffold_277.160	1	0.459	0.287	0.171	0.674
PSMB9	evm.model.scaffold_14.1201	1	0.276	0.121	0.155	1.19
HLA-DQB2	evm.model.scaffold_14.1074	16	0.315	0.167	0.148	0.915
C6orf136	evm.model.scaffold_277.185	1	0.501	0.371	0.13	0.434
GTF2H4	evm.model.scaffold_277.201.1	19	0.186	0.0812	0.105	1.2
DHX16	evm.model.scaffold_277.184	23	0.133	0.0638	0.0689	1.06
VARS2	evm.model.scaffold_277.200	35	0.229	0.166	0.0633	0.466
NELFE	evm.model.scaffold_353.210	3	0.158	0.0969	0.0616	0.71
ABCF1	evm.model.scaffold_277.190	7	0.146	0.0906	0.055	0.684
HLA-DRB5	evm.model.scaffold_14.1102	5	0.17	0.12	0.0497	0.501
ATF6B	evm.model.scaffold_353.196	6	0.225	0.183	0.0419	0.297
TAP1	evm.model.scaffold_14.1200	25	0.185	0.144	0.0406	0.358
DDX39B	evm.model.scaffold_353.218	4	0.228	0.192	0.0365	0.252
ATAT1	evm.model.scaffold_277.186.2	13	0.051	0.023	0.0279	1.15
BRD2	evm.model.scaffold_14.1096.4	20	0.0338	0.0113	0.0225	1.58
PPP1R10	evm.model.scaffold_277.189	8	0.109	0.0932	0.0159	0.228
C2	evm.model.scaffold_353.207	1	0.0159	0	0.0159	23.9
CFB	evm.model.scaffold_353.207	1	0.0159	0	0.0159	23.9
NRM	evm.model.scaffold_277.192	1	0.0156	0	0.0156	23.9
AIF1	evm.model.scaffold_353.227	3	0.0156	0	0.0156	23.9
DXO	evm.model.scaffold_353.213.6	20	0.0977	0.0857	0.0119	0.188
TRIM39-RPP21	evm.model.scaffold_277.175	37	0.0474	0.0371	0.0103	0.353
NEU1	evm.model.scaffold_353.216	16	0.0647	0.0559	0.00879	0.211
SKIV2L	evm.model.scaffold_353.211	8	0.272	0.292	-0.0201	-0.103
BAG6	evm.model.scaffold_353.224	7	0.298	0.32	-0.022	-0.103
EHMT2	evm.model.scaffold_353.205	6	0.14	0.168	-0.0274	-0.257
PRRC2A	evm.model.scaffold_353.226.5	17	0.221	0.254	-0.033	-0.201
PPT2	evm.model.scaffold_353.200	4	0.32	0.454	-0.134	-0.504
SLC44A4	evm.model.scaffold_353.203	4	0.206	0.366	-0.16	-0.828
PRRT1	evm.model.scaffold_353.198	1	0	0.387	-0.387	-28.5
